# Identification and pathogen screening of ectoparasites from companion animals in urban Vientiane, Lao PDR

**DOI:** 10.1371/journal.pntd.0013625

**Published:** 2025-10-15

**Authors:** Vanheuang Phommadeechack, Sungsit Sungvornyothin, Wirichada Pan-Ngum, Watthana Theppangna, Koukeo Phommasone, Elizabeth A. Ashley, Narisara Chantratita, Matthew T. Robinson, Rutcharin Potiwat

**Affiliations:** 1 Department of Medical Entomology, Faculty of Tropical Medicine, Mahidol University, Bangkok, Thailand; 2 Lao-Oxford-Mahosot Hospital-Wellcome Trust Research Unit, Microbiology Laboratory, Mahosot Hospital, Vientiane, Lao People's Democratic Republic; 3 Department of Tropical Hygiene, Faculty of Tropical Medicine, Mahidol University, Bangkok, Thailand; 4 Mahidol-Oxford Tropical Medicine Research Unit, Faculty of Tropical Medicine, Mahidol University, Bangkok, Thailand; 5 Department of Livestock and Fisheries, National Animal Health Laboratory (NAHL), Ministry of Agriculture and Forestry, Vientiane, Lao People's Democratic Republic; 6 Nuffield Department of Medicine, Centre for Tropical Medicine and Global Health, University of Oxford, Oxford, United Kingdom; 7 Department of Microbiology and Immunology, Faculty of Tropical Medicine, Mahidol University, Bangkok, Thailand; Oregon State University College of Veterinary Medicine, UNITED STATES OF AMERICA

## Abstract

Ticks and fleas are vectors of medically important infectious diseases globally, such as Rickettsiae. These pathogens are frequently reported in Southeast Asia, including Laos; however, there are very few comprehensive reports on their prevalence and vector diversity in urban areas. This study collected ectoparasites from companion animals to assess pathogen prevalence and exposure risk. In five veterinary clinics across Vientiane capital, ectoparasites were collected from dogs and cats and identified to the species level using both morphological and molecular methods. Ectoparasite DNA samples were screened for bacteria (*17-kDa* and *16S rRNA* gene). Ticks were submitted to evaluate the potential of MALDI-TOF mass spectrometry for species identification. A total of 3,771 arthropod vectors (3,658 ticks, 105 fleas, 8 lice) were removed from dogs and cats. Ticks were morphologically identified as *Rhipicephalus sanguineus* sensu lato (s.l.) tropical lineage (currently recognised as *Rhipicephalus linnaei*), whilst fleas were classified as either *Ctenocephalides felis felis* (57.1%) or *C. f. orientis* (42.9%) and lice were *Heterodoxus spiniger*. The MALDI-TOF spectra in this study revealed similar mass-to-charge (m/z) peak profiles to those reported in previous studies for *Rhipicephalus sanguineus*. *Rickettsia* spp. (*Rickettsia asembonensis* and *Rickettsia felis*) were detected in 44.4% of pooled flea samples collected from 12 dogs and 4 cats, as well as 3.5% of tick pools collected from 142 dogs and 50% of lice pools collected from 2 dogs. In addition, Anaplasmataceae (*Ehrlichia canis* and *Anaplasma platys*) were detected in 22.5% of ticks collected from dogs. This study highlights the diversity of ectoparasite species collected from dogs and cats and provide preliminary insights into the use of MALDI-TOF MS for tick species identification. While promising, further research is needed to enhance the reliability and efficacy of this approach. The findings also reveal a high prevalence of pathogens in ectoparasites, emphasizing the need for increased awareness among pet owners, veterinarians, and addressing public health concerns.

## Author summary

Ectoparasites such as ticks and fleas are commonly found on domestic animals and can carry pathogens that cause disease in both animals and humans. In this study, we collected 3,658 ticks, 105 fleas and 8 lice from dogs and cats from five veterinary clinics across Vientiane Capital, Laos and identified them using morphological characteristics, molecular techniques, and evaluated the potential of MALDI-TOF mass spectrometry (MS) for species identification of ticks. Ticks were identified as *Rhipicephalus sanguineus* s.l. tropical lineage (currently recognised as *Rhipicephalus linnaei*), fleas were classified into two subspecies: *Ctenocephalides felis orientis* and *C. f. felis*, while lice were identified as *Heterodoxus spiniger*. The ticks MS results showed spectral profiles similar to those reported for *Rh. sanguineus* in previous studies. *Rickettsia* spp. (*R. asembonensis* and *R. felis*), agents causing human diseases, were detected in 44.4% of flea pools collected from dogs and cats, as well as 3.5% of tick pools collected from dogs, and 50% of lice pools collected from dogs. Anaplasmataceae (*Ehrlichia canis*, *Anaplasma platys*) were detected in 22.5% of ticks collected from dogs. The findings revealed a high prevalence of pathogens in ectoparasites, particularly in fleas, indicating a possible risk of transmission to humans and animals living in close contact with them.

## Introduction

Ectoparasites are often medically important arthropod vectors that play a significant role in disease transmission in many countries and environments. Many of these diseases are zoonotic, being transmitted between animals and humans via a variety of transmission mechanisms associated with arthropod vectors [1]. Two of the most important groups of ectoparasites are ticks and fleas, which are commonly found in companion animals, wildlife and the environment [2,3]. Rickettsiae are a group of zoonotic bacteria that are commonly reported in ectoparasites, including ticks and fleas, and cause a variety of infectious diseases in many countries, especially in South and Southeast Asia regions such as India, Thailand, Myanmar, Cambodia and Laos [4–7].

In Lao P.D.R. (Laos), several studies have described rickettsial infection. In patients admitted to Mahosot Hospital, Vientiane, 27% had evidence of rickettsial infection including 14.8% with *Orientia tsutsugamushi* (causative agent of scrub typhus), 9.6% with *Rickettsia typhi* (murine typhus) and 2.6% with spotted fever group rickettsiosis (including *R. felis*, *R. helvetica* and *R. conorii*) [8]. Rickettsial infections have also been reported from patients in the north and south of Laos, with 7% of non-malarial febrile patients with scrub typhus infections, and less than 1% for those with murine typhus, *R. felis* or undetermined *Rickettsia* spp. [9]. Slightly lower prevalences were reported by Mayxay et al. in the south of the country, with 2.6% of non-malarial febrile patients with scrub typhus, 0.9% with Spotted fever group rickettsioses (SFGRs) and 0.4% with murine typhus infection [10].

Ticks and fleas are hematophagous ectoparasites of vertebrates, such as dogs, cats, rats and cows [2,3]. Because of their hematophagous life-style, a number of species of ticks and fleas have also implicated as vectors of various rickettsioses, including SFGRs such as the flea-borne spotted fever or cat-flea typhus (*Rickettsia felis*), *R. japonica*, *R. tamurae*, and *R. asembonensis* [11–15]. This group of pathogens can infect animals and humans through the bite of an infected ectoparasite, or contamination of a bite site or injury by the fecal material of an infected vector, or a crushed vector itself, depending on the type and species of vectors. Infection in humans is usually non-specific, generally causing high fever, headache, myalgia, and rash [12].

Given the importance of ectoparasites such as ticks and fleas in the transmission of vector-borne zoonoses that can cause febrile illness, it is essential to investigate their diversity and associated pathogens [7,12]. In Laos, particularly in urban areas like Vientiane Capital, there are very few comprehensive reports on ectoparasite diversity and pathogen prevalence, despite close human–animal interactions, and the country’s location within a region recognized as a hotspot for emerging infectious diseases. Several studies in Laos have identified ectoparasites carrying clinically relevant pathogens. *Rickettsia* spp. were identified in 5.7% of tick pools collected directly from the environment and companion animals in Khamouane Province (central Laos), including *R. tamurae, R. japonica* and other *Rickettsia* spp. isolates, and a further 2.3% likely positive for *Rickettsia* spp. Additionally, other relevant non-rickettsial pathogens were also identified, including *Borrelia* spp. in 1.6% pools, *Ehrlichia* spp. in 1.6%, *Coxiella* spp. in 1% and *Anaplasma* spp. in 0.3% pools [16]. Whilst this study looked at free-ranging vectors, two studies from northern Laos have looked at vectors collected from domesticated dogs. Kernif *et al*., collected fleas from domesticated dogs in Luang Namtha Province, and detected *Rickettsia felis* in 76.6% of fleas and *Bartonella* spp. in 3.3% [17]. Calvani *et al*., identified Rickettsiae in fleas collected from both cats and dogs, with *Rickettsia* spp. (including *R. felis*) detected in 100% and *Bartonella* spp. in 33.3% of flea samples [18]. A recent study reported the presence of two members of the family Anaplasmataceae, *Candidatus* Anaplasma pangolinii and an *Ehrlichia* spp., in 43.7% of pangolin ticks collected from pangolins in Vietnam and Laos [19]. Nguyen *et al*, collected vectors from dogs in the urban area of Vientiane capital. Over 86% of flea pools were positive for *Rickettsia* spp. and 100% were positive for Anaplasmataceae species (likely *Wolbachia* spp.). In ticks, 14.3% were positive for *Anaplasma* spp. (two pools confirmed as *A. platys*), 6.7% were positive for *Rickettsia* spp. and all of lice pools (two) were positive for *Rickettsia* spp. Of the *Rickettsia* spp. positives, 46% were identified as *R. felis* [20]. The previous studies highlight the prevalence of clinically relevant pathogens identified in ectoparasites found on companion animals and in the environment in Laos, which may pose a possible danger of disease exposure to pets and people. Whilst the majority of studies have been done in rural regions of the country, with a small number in urban areas, there is no information on actual pathogen exposure to humans in urban areas with a high proportion of companion animals (dogs and cats) and a high population density in the country [20].

Identifying arthropod vectors is crucial for comprehending epidemiology, monitoring epidemics, and planning vector control programs. However, there is a challenge in morphologically identifying the species of a large number of individuals, which is a time-intensive process, as well as limitation in availability of entomological expertise and dichotomous keys. In addition, the engorged/unengorged status and immature life stages (particularly of ticks) may provide difficulties during morphological identification [21]. Molecular identification is essential to efficiently describe biodiversity of ectoparasites and has been used as an alternative to overcome the limitations of morphological identification. The mitochondrial cytochrome c oxidase subunit 1 (*COI*), a fragment of about 500–800 base pairs from mitochondrial DNA, is commonly used as the standard marker for DNA barcoding [21,22]. In Laos, few studies describe the genetic identification and divergence among ectoparasites, indicating the potential benefits of implementing molecular tools to support morphological identifications [18]. However, molecular identification is laborious, expensive, time-consuming and difficult to apply for species for which sequences are not available [21]. Previous studies have been shown the potential of matrix-assisted laser deionization time-of-flight mass spectrometry (MALDI-TOF MS) to allow high throughput identification of vectors, using small amounts of tissue, for a relatively low cost (when used as high-throughput). Studies have shown a concordance rate of 99.6% in tick identification using MALDI-TOF MS in comparison to morphological and molecular methods, and suggested a potential application of MALDI-TOF MS for species identification on ticks obtained from domestic animals and cattle [21].

In this study, arthropod ectoparasites were collected from dogs and cats in Vientiane capital. Identification of the vectors was done using morphological and molecular methods, along with molecular methods to determine any pathogen carriage. In addition, the present work evaluated the potential of the MALDI-TOF (BioMerieux, France) mass spectrophotometer to identify the species of ticks found on dogs in Laos.

## Methodology

### Ethics statement

Animal ethical clearances, including the protocol, informed consent form, PIS and CRF were approved from the Institutional Review Board of the Department of Fisheries and Livestock of Laos (document no. 2526/DLF.IRB) and The Animal Care and Use Committee of the Faculty of Tropical Medicine, Mahidol University (certificate no. FTM-ACUC 005/2023E). In addition, the protocol was granted by Mahidol University Institutional Biosafety Committee (approval no. MU 2023–010).

### Sample collection

Sample collection was carried out at five veterinary centres between March to June 2023 in Vientiane city, Lao PDR. Dogs and cats being brought into the veterinary surgery for any reason were checked for ectoparasites by the attending veterinarian. Following consent from the owner, participant information sheet (PIS) and case record form (CRF) were obtained from the participant. Ectoparasites found on the animals were removed by the veterinarian and preserved in 70% ethanol (v/v). All specimens were transferred to the Lao-Oxford-Mahosot Hospital-Wellcome Trust Research Unit (LOMWRU) laboratory.

### Morphological and molecular identification

Arthropods were identified at genus and species level, including life stage and sex, based on species morphological characteristics and dichotomous keys previously published [2,3,23–26]. Arthropod samples were pooled by host, species, feeding status, life stage and sex (for adult stages only), and ranged from 1 to a maximum of 10 individuals per pool (with multiple pools possible per host). DNA was extracted following the tissue extraction protocol from GeneJET Genomic DNA purification kit (Thermo Scientific, USA), with the following adapted process: vector samples were cut into small pieces and homogenized in 180 μL of digestion buffer; 20 μL of Proteinase K was added, followed by overnight incubation at 56°C. DNA samples were eluted twice in 50 μL volumes for a final volume of 100 μL. An aliquot of DNA was transferred to the Medical Entomology Department laboratory, Faculty of Tropical Medicine Mahidol University, for molecular identification targeting *COI* gene (LCO1490 and HCO2198) as described previously [18,27,28] (see S1 File). The COI-derived nucleotide sequence was submitted to the NCBI GenBank database. A phylogenetic tree was constructed to compare the related species of ectoparasites.

### MALDI-TOF identification

To determine the suitability of using MALDI-TOF identification in Laos, a subset of 79 individual ticks were rinsed in 70% ethanol, followed by distilled water and then dissected to separate all four legs from the same side of ticks using a sterile surgical blade. The protein extract was obtained by using the adapted procedures outlined in earlier investigations [21,29–33]. Briefly, tick legs were manually homogenised in 15 μL of 70% formic acid and 15 μL of 50% acetonitrile. The mixture was then incubated at room temperature for 5 minutes, followed by centrifugation at 2000 xg for 30 seconds to pellet debris. One microlitre of the homogenised supernatant from each sample was placed onto the MALDI-TOF target plate spot in triplicate (Biomerieux, France). Then, 1 μL of MS-CHCA matrix (VITEK, BioMérieux) solution was added to all samples; a sample spot with CHCA matrix-only served as control. The MALDI-TOF target slide was analysed in Vitek MALDI-TOF MS device (BioMerieux, France) using research user only (RUO) mode. The protein mass profile was generated in a mass range of 2–20 kDa (2,000–20,000 m/z) with detection in the linear positive-ion mode at a laser frequency of 50 Hz [21,29–33]. Protein mass profile was obtained from BioMérieux platform SARAMIS Premium (BioMerieux, France).

### Pathogen detection

Ectoparasite DNA, including individual or pooled samples were screened for endemic zoonotic pathogens with specific primers. Based on the findings of the previous study [20], real-time PCR targeting *Rickettsia* spp. *17-kDa* gene [34], modified to use PrimeTime master mix (PrimeTime, Integrated DNA Technologies, USA), and conventional PCR targeting the *16S rRNA* gene of Anaplasmataceae [19,35] were used. Samples testing positive for *Rickettsia 17-kDa* gene were further analyzed using nested PCR (nPCR) targeting *17-kDa* gene [16]. PCR conditions are provided in S1 File. PCR product from Anaplasmataceae and *Rickettsia* nPCR positive samples were purified using the GeneJET Gel Extraction kit (Thermo Scientific, USA) and sent to Macrogen (South Korea) for Sanger sequencing analysis. The sequences analysis were processed using Bioedit version 7.2.5, merged for alignment with ClustalX version 2.1, and the sequence was submitted to the BLAST system on NCBI to compare the sequence with the reference material in the GenBank database. The genetic comparison sequencings were acquired from the NCBI database with the accession number. Estimated prevalence rates of individual vectors per 1,000 were calculated using the PooledInfRate program along with 95% confidence intervals [36]. In addition the minimum possible infection rate (MinIR) and the maximum infection rate (MaxIR) was calculated to provide a possible range of pathogen infection rates in ectoparasites, assuming at least one infected individual per positive pool (MinIR), or that all individuals were infected in any positive pools (MaxIR).

## Result

Throughout the enrolment period, a total of 282 dogs and 25 cats were recruited and examined for the presence of vectors (S1 Data). In total, 3,771 arthropods, including ticks, fleas and lice, were collected from 151 of dogs and four of the cats. Of the total animal enrolment, 69.3% (104/150) of dogs and all cats were healthy, 30.6% (46/150) of dogs were indicated to have an illness during the enrolment period, and 28.6% (43/150) reported an illness in the last 7 days (Table 1). A total of 3,653 ticks were removed from 151 dogs with the median of 9 per dog (inter quartile range or IQR 4.0-22.7, range 1–330), and five ticks were removed from a cat. Of 105 fleas, 82 were collected from 12 dogs (78.1%), with a median number of 2 per dog (IQR 2.0 -7.0, range 1–31) and 23 from four cats (21.9%), with a median of 5 per cat (IQR 3.5-7.3, range 2–11). Eight lice were obtained from two dogs (median 4/dog, IQR 2.5-5.5, range 1–7) (Table 1). Whilst the majority of dogs had only one type of vector, ten dogs (6.6%) were reported with both ticks and fleas, one dog with both ticks and lice, and one dog was reported with all three of these arthropods. In addition, one cat was reported with both ticks and fleas.

**Table 1:** Demographic and clinical characteristics of dogs and cats.

Characteristics		Dog	Cat
*n*		150*	4
Median age (IQR, range)		1.3 (0.5–3.0, 0.1–19)	0.8 (0.6–1.5, 0.4–3)
Sex, n (%)	Male	82 (54.3)	1 (25)
	Female	69 (45.7)	3 (75)
Last 7 days illness, n (%)	Yes	43 (28.6)	0 (0)
	No	107 (71.3)	4 (100)
Current illness, n (%)	Yes	46 (30.6)	0 (0)
	No	104 (69.3)	4 (100)
Median number of vectors found on animals (IQR, range)	Tick	9 (4.0–22.7, 1–330)	5 (5.0–5.0, 5–5)
	Flea	2 (2.0–7.0, 1–31)	5 (3.5–7.3, 2–11)
	Lice	4 (2.5–5.5, 1–7)	0 (0)

IQR, inter quartile range; n, number of hosts.

*Total number of dogs 151, 150 had clinical details, 130 had age data.

### Species identification of ectoparasites

#### Morphological identification.

All ticks collected from dogs and cats were morphologically identified as *Rhipicephalus sanguineus* s.l.; the percentage of developmental stages were composed of 35.5% adult males, 36.7% adult females, 22.1% nymphs, and 5.7% larvae. The male to female adult tick ratio was 1:1.03. Ticks were classified by blood feeding status during specimen collection as blood fed (including partially blood-fed and fully engorged individuals) or unfed status. Of 3,653 ticks collected from dogs, 23.8% were blood fed adult females, 12.9% were unfed adult females, 15.7% were blood fed nymphs, 6.4% were unfed nymphs, 5.2% were blood fed larvae, and 0.5% were unfed larvae. Feeding status of adult males could not be determined (Table 2). From the one cat identified with 5 ticks, the number of developmental stages was composed of 1 adult male, 1 adult unfed female, 2 unfed nymphs, and 1 fed nymph.

**Table 2:** Number of Ticks from dogs based on life stages.

Tick life stage	Blood fed n (%)	Unfed n (%)	Total number n (%)
Larva	189 (5.2)	19 (0.5)	208 (5.7)
Nymph	573 (15.7)	232 (6.4)	805 (22.1)
Adult female	871 (23.8)	471 (12.9)	1,342 (36.7)
Adult male*			1,298 (35.5)

*Feeding status of adult male not determined

From dogs, two species of fleas were morphologically identified from 82 specimens: *Ctenocephalides felis felis* (45.1%) and *Ctenocephalides felis orientis* (54.9%). The proportion of *C. f. felis* were 13.4% males, and 31.7% females. The male to female flea ratio was 1:2.36. The percentages of *C. f. orientis* were 15.9% males and 39.0% females, with a 1:2.46 male to female ratio (Table 3). The fleas identified on cats included 30.4% (7/23) of male *C. f. felis* and 69.6% (16/23) of female *C. f. felis*, with 1: 21.28 male to female ratio (Table 3). All of the lice found on dogs were identified as *Heterodoxus spiniger*, and comprised of 75% of male *H. spiniger* and 25% of female *H. spiniger*.

**Table 3:** Number of fleas identified on dogs and cats.

Species	Sex	Dog n (%)	Cat n (%)
*C. f. felis*	Male	11 (13.4)	7 (30.4)
	Female	26 (31.7)	16 (69.6)
	M: F ratio	1: 2.36	1: 2.28
*C. f. orientis*	Male (M)	13 (15.9)	
	Female (F)	32 (39.0)	
	M: F ratio	1: 2.46	

#### Molecular identification.

40 DNA samples consisting of 33 pooled tick samples, five pooled flea samples, and two pooled lice samples, were examined for the *COI* gene by PCR. All samples provided a positive result with expected band at 650 base pairs. A subset of PCR products were sent for sequencing to confirm morphological identification. Pooled tick samples showed 100% similarity to *Rh. sanguineus* s.l. from Colombia (KT906182), Angola (MF425994), Vietnam (PP389595) and China (OQ704652, OQ704663, OQ704678, JQ737084), confirming morphological identification. Specimens of *C. f. felis* had 97-99.8% similarity to *C. f. felis* from India (KX467335) and Thailand (OQ291320), whilst *C. f. orientis* specimen had 99.8% similarity to *C. f. orientis* from Thailand (OQ291337). A louse specimen was 97.9-99.5% identical to *H. spiniger* from South East Asia regions (MT027225) and Saint Kitts and Nevis (OQ779697) (S1 Table). The representative sequences of each ectoparasite species found on dogs and cats were submitted to the GenBank data base under accession numbers PV341327-PV341341 for ticks and PV341342-PV341345 for fleas (see S2 Table).

**Fig 1:**
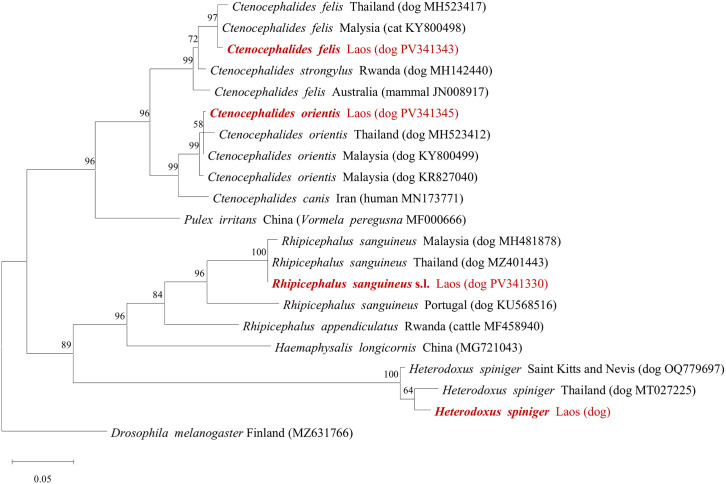
Phylogenetic tree analysis of *COI* gene sequence of ectoparasites including ticks, fleas and lice collected from dogs, with the comparison of *COI* gene sequence obtained from NCBI GenBank. Phylogenetic were construct by maximum likelihood analysis using MEGA11, with 1,000 number of Bootstrap replicates. Percent homology of ectoparasite indicates as the number in the branch points.

Phylogenetic tree of the partial *COI* gene of the representative ectoparasites collected from dogs were analyzed using MEGA11, with the comparison of the sequence obtained from the NCBI GenBank, rooted by *Drosophila melanogaster* (MZ631766). The representative *COI* gene sequences of ectoparasites in this study, including *Rh. sanguineus* s.l. (P004-T1), *C. f. felis* (P008-F1), *C. f. orientis* (P018-F1), and *H. spiniger* (P031-L1), showed a different genetic clade compared to the sequence obtained from GenBank (Fig 1). *C. f. felis* was more closely related to *C. f. felis* obtained from dogs in Malaysia (KY800498) and *C. f. felis* from stray dogs in Thailand (MH523417) than *C. f. felis* collected from red fox in Australia (JN008917). *C. f. orientis* sharing clade with *C. f. orientis* collected from dog in Thailand (MH523412) and Malaysia (KY800499, KR827040). *Rh. sanguineus* s.l. collected from dog in this study and *Rh. sanguineus* collected from dogs in Malaysia (MH481878) and Thailand (MZ401443) were more closely related to each other. *H. spiniger* was genetically closer to *H. spiniger* collected from a dog in East- and Southeast Asia region (MT027225) (i.e., Vietnam, Philippines, Thailand, China, Taiwan).

#### MALDI-TOF MS evaluation.

Of the 79 tick leg samples analyzed by MALDI-TOF MS, good quality spectra were obtained from 14 tick leg samples, either preserved at -20°C (26.09%) or preserved in 70% EtOH (73.91%). Protein analysis was conducted in the range of 2–20 kDa. Key peaks of mass-to-charge ratio (m/z) were identified at around, 4,023 (4,023–4,024), 4,088 (4,088-4,089), 6,152 (6,151–6,154), 8,047 (8,045–8,049), 11,492 (11,477–11,588) and 12,292 (12,217–12,292) Daltons (Da). Spectra profiles were similar to spectra published by other studies [21,30–33], with some limited variation. Published *Rh. sanguineus* profiles indicated key identifying peaks at 4,020, 6,082, 8,042, 11,500, and 12,207 Da (S1 Fig).

### Pathogen detection

PCR screening for bacterial pathogens was conducted on 296 pools of ticks from 142 dogs, 3 pools of ticks from a cat, 15 pools of fleas from 12 dogs, 4 pools of fleas from 4 cats, and 2 pools of lice from 2 dogs (Table 4). Among pooled samples from dogs, 13.8% (41/296) of tick pools were positive for Anaplasmataceae, and 1.7% (5/296) were positive for *Rickettsia* spp. Of the Anaplasmataceae, 21.9% were identified as *Ehrlichia canis*, with 97.6-100% similarity (accession no MN922610, KT357374), 39.0% as *Anaplasma platys* (96.7-100% identity, accession no CP046391, KT359590, MN922608, KU586028, LC126863) and two pools (4.9%) with 91.9-94.2% similarity to *R. asembonensis* (accession no MN003387, MK923744). In addition, *Rickettsia* spp. were detected in 11.1% of *C. f. felis* and 83.3% of *C. f. orientis* pooled samples. One *C. f. felis* pooled sample was 100% match to *R. felis* (accession no MK509750.1). *Rickettsia* spp. detected in all of *C. f. orientis* pooled samples were 99.8-100% similarity to *R. asembonensis* (accession no MK923744). One of the 2 lice pools was positive for *R. asembonensis* with 91.7% identity (accession no MK923744) (Table 4). Relating the positive pools to host animals, 22.5% of dogs with ticks (32/142) had at least one tick pool positive for Anaplasmataceae (*E. canis*, *A. platys*), and 3.5% positive for *Rickettsia* spp. Lice from one dog was confirmed positive for *R. asembonensis* (Table 5).

**Table 4:** Bacteria detection in ectoparasite samples based on individual or pooled samples.

Ectoparasites	Pathogens	Dogs % (number/total)	Cats % (number/total)
*Rh. sanguineus* s.l.	Genus *Rickettsia*	1.7 (5/296)	0 (0/3)
	Family Anaplasmataceae	13.8 (41/296)	0 (0/3)
	*Ehrlichia canis*	21.9 (9/41)	
	*Anaplasma platys*	39.0 (16/41)	
	*R. asembonensis*	4.9 (2/41)	
*C. f. felis*	Genus *Rickettsia*	11.1 (1/9)	50 (2/4)
	*R. felis*	11.1 (1/9)	25 (1/4)
	Family Anaplasmataceae	100 (9/9)	100 (4/4)
	*Wolbachia* spp.	33.3 (3/9)	50 (2/4)
*C. f. orientis*	Genus *Rickettsia*	83.3 (5/6)	
	*R. asembonensis*	83.3 (5/6)	
	Family Anaplasmataceae	83.3 (5/6)	
	*Wolbachia* spp.	16.6 (1/6)	
*H. spiniger*	Genus *Rickettsia*	50 (1/2)	
	*R. asembonensis*	50 (1/2)	

**Table 5:** Number of dogs and cats infested with ectoparasites and identified with bacteria.

Vector species	% hosts with positive vectors (number/total tested)			
	*Rh. sanguineus* s.l.	*C. f. felis*	*C. f. orientis*	*H. spiniger*
Dog (n = 142)				
*Rickettsia* spp. (q/nPCR)	3.5 (5/142)	11.11 (1/9)	100 (5/5)	50 (1/2)
*R. asembonensis*			100 (5/5)	50 (1/2)
*R. felis*		11.11 (1/9)		0 (0/2)
Anaplasmataceae (cPCR)	22.5 (32/142)	100 (9/9)	100 (5/5)	0 (0/2)
*E. canis*	3.5 (5/142)			0 (0/2)
*A. platys*	11.2 (16/142)			0 (0/2)
*R. asembonensis*	1.4 (2/142)			0 (0/2)
*Wolbachia* spp.	0 (0/142)	33.3 (3/9)	20 (1/5)	0 (0/2)
Cat (n = 4)				
*Rickettsia* spp. (q/nPCR)	0 (0/1)	50 (2/4)		
*R. felis*		25 (1/4)		
Anaplasmataceae (cPCR)	0 (0/1)	100 (4/4)		
*Wolbachia* spp.		50 (2/4)		

Among pooled samples from cats, 50% of fleas (*C. f. felis*) were positive for *Rickettsia* spp., with one sample confirmed as *R. felis* by sequencing (accession no MK509750). There were no positive result from ticks found on cats. In addition, 100% of *C. f. felis* pooled samples collected from dogs and cats, and 83.33% of *C. f. orientis* pooled samples collected from dogs were positive for Anaplasmataceae. Sequencing these as *Wolbachia* spp. in 50% and 33.3% of *C. f. felis* (collected from cats and dogs, respectively), and 16.6% of positive *C. f. orientis* collected from dogs (accession no CP051156) (Table 4). A total of five dogs had *C. f. orientis* positive for *R. asembonensis*, one of them also had fleas positive for *Wolbachia* spp., whilst 11.1% (1/9) dogs with *Wolbachia*-positive *C. f. felis* pools were also positive for *R. felis*. Of four cats identified with *C. f. felis*, one had fleas positive for *R. felis* (Table 5).

**Fig 2:**
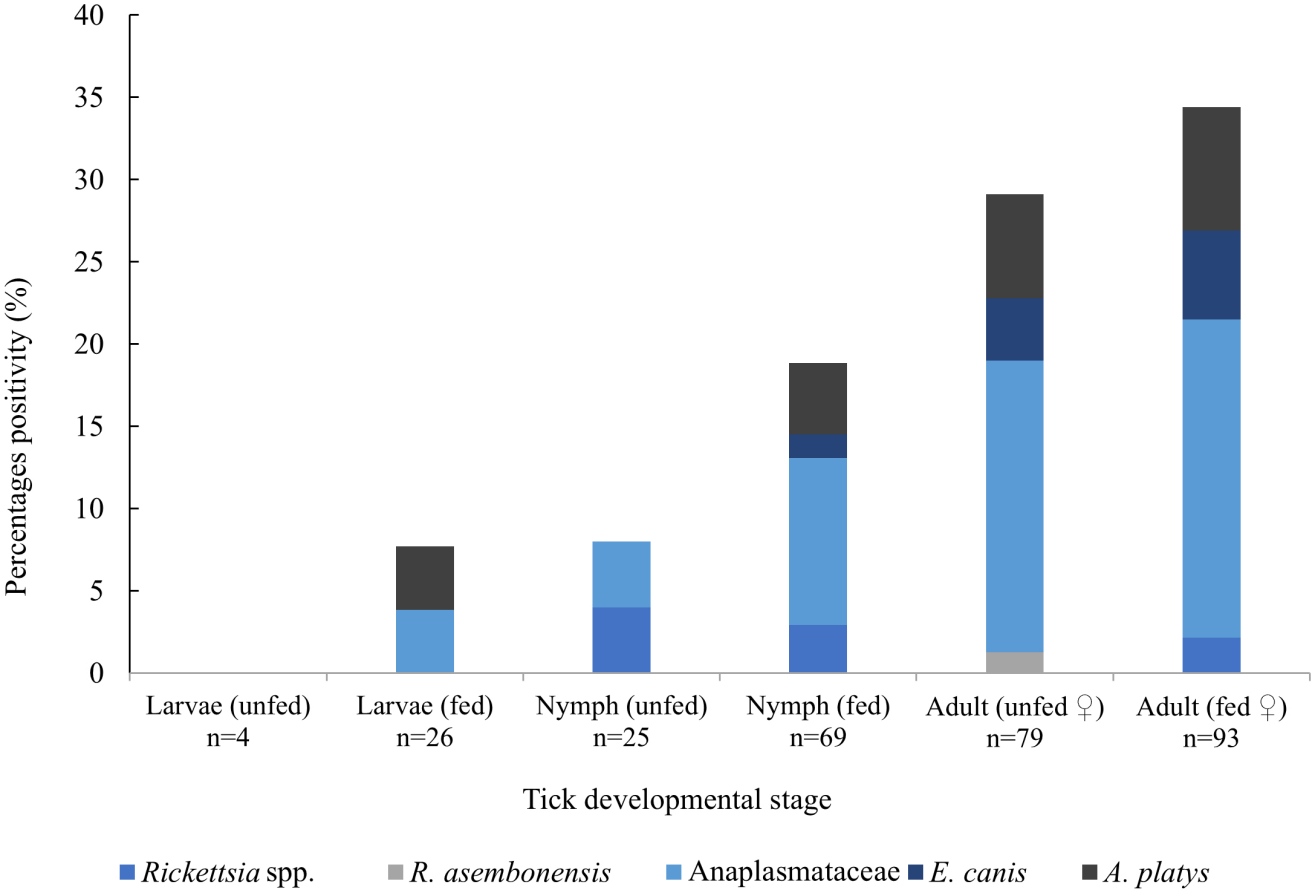
Bacteria detection in *Rh. sanguineus* s.l. collected from dogs based on the life stage, feeding status and sex.

**Fig 3:**
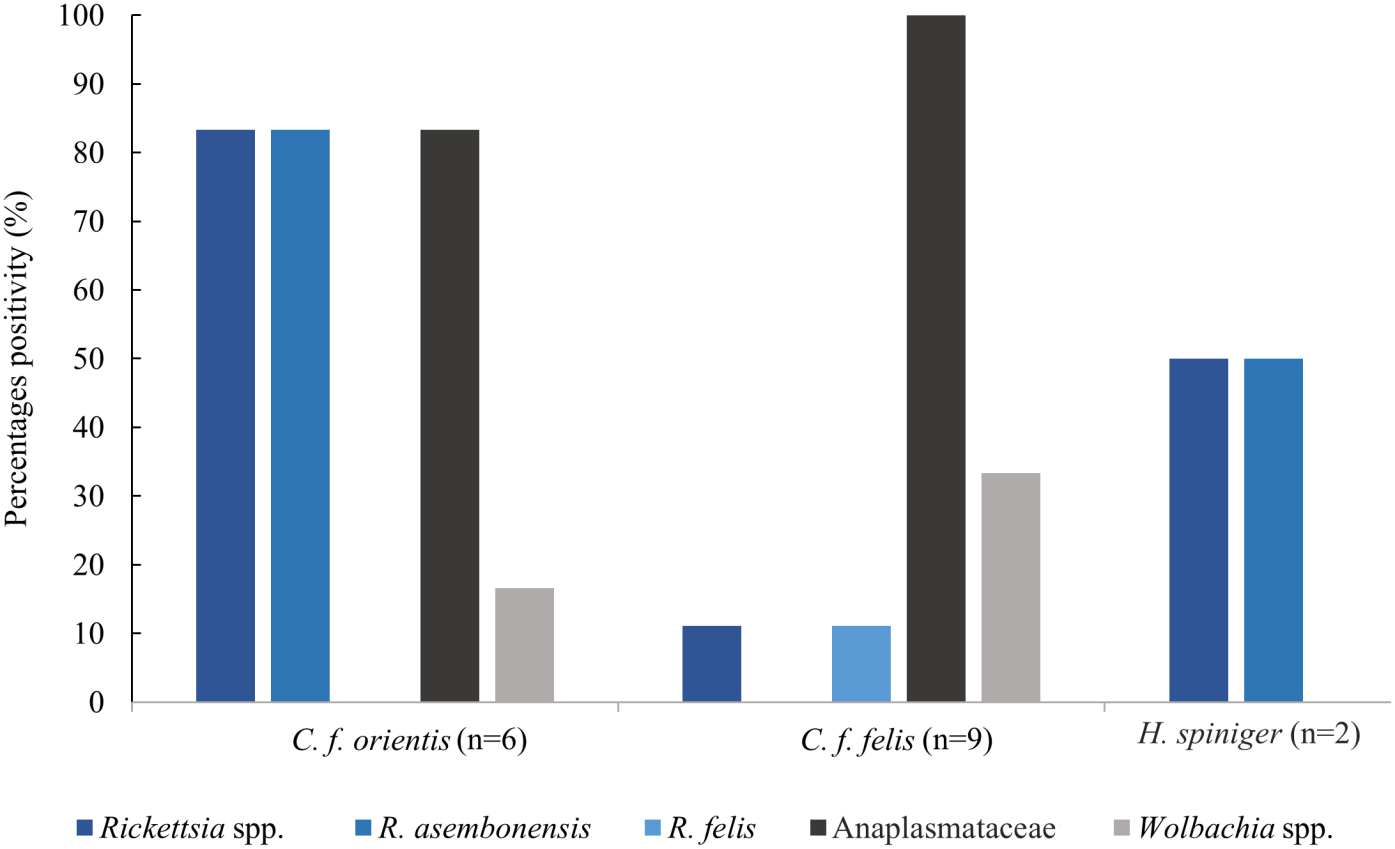
Bacteria detection in fleas and lice collected from dogs.

**Fig 4:**
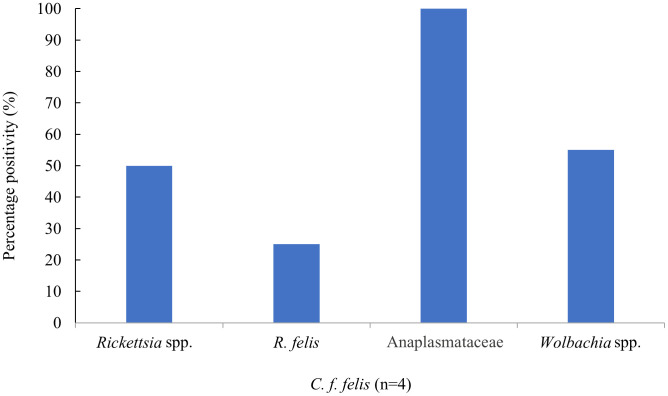
Bacteria detection in *C. f. felis* collected from cats.

*Rickettsia* spp. in ticks was detected in 4% (1/25) of unfed nymphs, 2.9% (2/69) of fed nymphs and 2.2% (2/93) of fed adult females. Anaplasmataceae were found in 3.8% (1/26) of fed larvae, 4% (1/25) of unfed nymphs, 10.2% (7/69) of fed nymphs, 17.7% (14/79) of unfed adult females and 19.4% (18/93) of fed adult females. There were no pathogens detected in unfed larvae, Fig 2. The distribution of pathogens found in fleas and lice collected from dogs and cats are described in Figs 3 and 4, respectively. Where possible, estimated prevalence rates in individual vectors was calculated along with 95% confidence intervals, MinIR and MaxIR. The minimum possible infection rate (MinIR) and the maximum infection rate (MaxIR) was calculated to provide a possible range of pathogen infection rates in ectoparasites. Infection rates for Anaplasmataceae in ticks from dogs (*E. canis* and *A. platys*) infection rate was estimated at 43.06 per 1,000 (95%CI 31.62-57.33; MinIR = 4%, MaxIR = 19.2%). *Rickettsia* spp. in fleas from dogs was estimated at 127.13 per 1,000 (95%CI 59.79-247.24; MinIR = 9.6%, MaxIR = 56.2%), whilst *Rickettsia* spp. in *C. f. felis* collected from cats was estimated at 102.9 per 1,000 (95%CI 22.49-323.39; MinIR = 10.0%, MaxIR = 45.0%) (S3 Table).

## Discussion

The presence of the causative agents of a number of clinically important in ectoparasites collected from animals and environment continues to pose a significant concern to animal and human health [16–18,20]. These pathogens have been reported in various regions of Laos, but mainly in the rural areas. This study confirms the distribution of vectors and the prevalence of pathogens in ectoparasites collected from dogs and cats in Vientiane, the capital city of Laos, a relatively densely populated urban area. The ectoparasite species collected from dogs and cats in this study are consistent with the diversity in species collected previously in Vientiane [20] and as described from dogs and cats in Northern Laos [18]. High prevalences of pathogens were seen, particularly in fleas collected from both dogs and cats. *Rickettsia* spp. (*R. asembonensis*, *R. felis*) were detected in 44.44% (8/18) of pooled fleas, collected from 12 dogs and four cats, with a possible infection rate in fleas ranging from 9.6% to 56.2%, based on possible minimum and maximum infection rates. *Rickettsia* spp. (*R. asembonensis, R. felis*) was detected in 100% (5/5) of *C. f. orientis* and 11% (1/9) of *C. f. felis* from dogs, with a possible minimum and maximum infection rate between 15.4% to 100% and 2.9% to 5.9%, respectively, and in 50% (2/4) of *C. f. felis* from cats, with 10% to 45% of minimum and maximum infection rates. Whilst the results suggests that there is a possibility of risk exposure to pathogens among companion animals and their owners, caution must be taken when basing interpretation on species of companion animal. The limited numbers of cats in this study being taken to veterinary surgeries may induce some bias in positivity data.

All ticks were identified as *Rh. sanguineus* s.l., a brown dog tick commonly found on dogs and previously described in the region [17,20]. As confirmed by sequencing, it is likely that these ticks are *Rh. sanguineus* s.l. tropical lineage (recently renamed as *Rh. linnaei* [37]). *Rh. sanguineus* is recorded as being highly suited to living in urban as well as rural areas, and is particularly adapted to living within human habitation, being active throughout the year in tropical and subtropical regions [38], and therefore its exclusive presence in this study is not unexpected. The fleas found on cats were identified as *C. f. felis*, while fleas found on dogs were both *C. f. felis* and *C. f. orientis*, corresponding to previous studies [18,20]. Whilst one study reports a higher incidence of *C. f. orientis* in dogs [17], this current study supports the results of by Calvani et al. [18], which indicated a higher proportion of *C. f. felis* compared to *C. f. orientis* among dogs and cats in the region using similar molecular identification (*COI* gene). There was no *C. canis* identified in this study, supporting previous reports [2,18,20,39]. The large number of ectoparasites collected, and therefore needing morphological identification, proved highly time-consuming and challenging at times. Therefore, identification at genetic level remains important to confirm and overcome the limitation of morphological identification. Correct morphological identification was confirmed by molecular identification through PCR and sequencing of the *COI* gene. Whilst there was a risk that pools might have accidentally contained different species of vectors, successful sequencing of PCR products indicated that this was not the case. Whilst molecular identification proved successful, it is still time consuming and resource intensive, particularly if ectoparasites cannot be pooled. This study described the initial information on the utilisation of the Maldi-TOF MS approach for use in species identification of ticks in Laos. The spectra for *Rh. sanguineus* s.l. obtained here showed similarity to key identification peaks obtained in previous studies [21,30–33]. Whilst only one species of tick was identified in this study and therefore trialed on MALDI-TOF, other studies have successfully differentiated between different species of ticks (such as *Rh. bursa* and *D. marginatus* [31]), and *Rh. sanguineus* infected with *R. conorii* [32]. Given that a proportion of the samples produced good peaks (14/79), further optimization on sample preparation is required to evaluate the use of MALDI-TOF in the Lao context, as this requires a high concentration of protein containing in the samples and appropriate condition for sample preservation. Inherent biological variations, such as feeding status and the presence of potential pathogens, may also influence the results [33]. Samples that were identified using all three methods gave matching species identifications, indicating that MALDI-TOF MS could be reliably used for high-throughput ectoparasite identification, once further optimization has been done. With no commercially available spectra database, immediate application of MALDI-TOF MS is limited until a region-specific database is built.

*Rickettsia* spp., a known genus of pathogens causing a variety of human rickettsiosis in Laos [8–10], was detected in ectoparasites collected from dogs and cats. *R. felis* was detected in 25% and 11% of fleas collected from cats and dogs, respectively. *R. felis*, is a known agent causing rickettsiosis [11] and has previously been reported in patients in Laos [8,9] as well as in ectoparasites including tick and fleas in rural and urban areas [17,18,20]. The *R. felis*-like organism *R. asembonensis* was found in 100% *C. f. orientis*, 50% *H. spiniger*, and in 1.4% ticks collected from dogs. *R. asembonensis* is seen as an agent with potential for human and animal infection, but with uncertain human pathogenicity at present [40] and is commonly found in fleas around the world, including South East Asia and neighboring country, such as Thailand [39,41,42]. In Laos, *R. asembonensis* was previously reported in 27.3% of *C. felis* collected from dogs [20]. This study reports *R. asembonensis* in two tick pools collected from dogs. There is little to no information regarding *R. asembonensis* in ticks, and may be due to ticks consuming an infected blood meal. Additional research is needed to confirm the possible implication of ticks in the *R. asembonensis* transmission cycle. *Wolbachia* spp. detected in all flea samples collected from dogs and cats is a known bacterial endosymbiont commonly found in fleas [20,43] but with no clinical implications for humans. However, its presence is noteworthy in a One Health context, as *Wolbachia* can influence vector biology and pathogen interactions, and has been considered in vector control strategies [44].

Pathogen detections were presented in each life stage and feeding status of tick (except for unfed larvae). Previous study reported pathogens (*Rickettsia* spp., *Ehrlichia* spp, *Borrelia* spp., *Anaplasma* spp., *Coxiella* spp.) in each life stage of tick collected from the environment and companion animals in the south of Laos [16]. Both Rickettsiae and Anaplasmataceae have been found to be transovarially and transtadially transmitted in ticks, therefore the presence of these pathogens in unfed ticks suggests that there is a possibility that pathogens could be transmitted to hosts (including humans) when feeding [3,45–47]. However, this should be further investigated to confirm the efficacy of pathogen transmission among ticks, and other vectors, and the risk of exposure for humans. There is a risk that pools will contain multiple pathogens which may be overlooked when relying on PCR identification. Using broad-range PCRs and sequencing of PCR products from pools in this study, indicated that this was not the case. In addition, pooled extraction of samples may result in inaccurate assessment of pathogen prevalence in assessing the risk of pathogens in individual vectors. Despite this, the minimum and maximum infection rates (IR) calculated in this study suggests there is potential for a high level of risk of exposure of humans to *Rickettsia* spp. from ectoparasite infested dogs and cats, especially in fleas, and highlights the need for raising awareness of adequate vector control with pets.

The detection of pathogens in ectoparasites collected from cats and dogs suggests a potential risk for subclinical or clinical infections in these animals. This highlights the importance of routine veterinary monitoring and ectoparasite control. Cats and dogs may act as reservoirs, and ectoparasites such as fleas and ticks serve as vectors. Consequently, humans in close contact with pets may be at risk of acquiring infections. These findings emphasize the importance of regular ectoparasite control, routine grooming, and vaccination in pets, along with public awareness campaigns, to reduce the risk of zoonotic transmission.

## Conclusion

This study updates and confirms the species identification of ectoparasites obtained from dogs and cats in Vientiane, and urban region of Lao PDR, using morphological and molecular identification. In addition, this study provides initial support for the use of MALDI-TOF MS tools for species identification of ectoparasites. The work describes the high prevalence of *R. felis* and *R. asembonensis* in ectoparasites, suggesting a risk of exposure for humans and other animals. Additional studies on the exposure risk of this pathogen in humans and animals is essential to enhance our understanding of its prevalence in the area. The findings indicate a high occurrence of bacteria that may cause illnesses in animals and their owners in urban areas, highlighting the need for increased awareness in owners to adopt regular ectoparasite control, routine grooming, and vaccination. In addition, public awareness of zoonotic risks should be raised, and the use of adequate vector control strategies in densly populated areas.

## Supporting information

S1 Table Morphological and molecular identification targeting *COI* gene of ectoparasites. ♂, adult male; ♀, adult female; L, larvae; N, nymph; b, blood fed; uf, unfed.
(XLSX)

S2 Table Accession numbers of ectoparasites found on dogs and cats submitted to the GenBank database. (XLSX)

S3 Table Infection rates. (XLSX)

S1 File Overview of PCR assays. (DOCX)

S1 Fig MALDI-TOF mass spectrometry identification from *Rh. sanguineus* s.l. collected from dogs. Grey dotted lines represent key identifying peaks from previously published *Rh. sanguineus* spectra.
(TIF)

S1 Data Raw data. (XLSX)
