# Supplementary material for: Identification and pathogen screening of ectoparasites from companion animals in urban Vientiane, Lao PDR
Source: PLoS Negl Trop Dis. 2025 Oct 15;19(10):e0013625. doi: 10.1371/journal.pntd.0013625 (PMC12543278; doi:10.1371/journal.pntd.0013625)
Supplement: S1 File — (DOCX) [file pntd.0013625.s004.docx]

| **S1 File.** **Overview of PCR assays** | | | | | |
| --- | --- | --- | --- | --- | --- |
| PCR target | Primer name /sequence (5' to 3') | Product size (bp) | Mastermix (rnx) | Thermocycler and detection | Reference |
| *COI* gene cPCR | LCO1490: GGTCAACAAATCATAAAGATATTGG  HCO2198: TAAACT-TCAGGGTGACCAAAAAATCA | 650 | The 50μL total reaction volume includes TaKaRa Ex TaqTM DNA polymerase (0.25μL), 10x buffer, 20 mM Mg^2+^ (5μL), 200mM dNTPs mix (4μL), 10μM primers (1μL each), H_2_O, DNA template (5μL) | 95°C 10 min  35 cycles of:  94°C 45 s, 55°C 45 s, 72°C  1 min  72°C 7 min (final extension)  2% agarose gel | [1,2,3] |
| *17-kDa* qPCR | R17K128F2: GGGCGGTATGAAYAAACAAG  R17K238R: CCTACACCTACTCCVACAAG  R17K202TAQP: FAM-CCGAATTGAGAACCAAGTAATGC- TAMRA |  | The 20μL total reaction volume includes 2x PrimeTime master mix (10μL), 10μM primers and probe (0.8μL each), H_2_O, DNA template (2μL) | 95°C 3 min,  45 cycles of:  95°C 15 s, 60°C 1 min, plate read | [4] |
| *17-kDa* nPCR | R17kM61F: ACTTTACAAAATTCTAAAAACCATATACT  Rr2608Rnew: CATTGTCCGTCAGGTTGGCG | 523 | The 25μL total reaction volume includes Platinum® Taq DNA polymerase (0.1μL), 10x PCR Buffer (2.5μL), 10mM dNTP mixture (0.5μL), 50 mM MgCl2 (1μL), 10 µM primers (0.75μL each), H_2_O, DNA template (1μL) | Primary PCR: 94°C 1 min  35 cycles of: 94°C 30 s, 55°C 30 s, 68°C 2 min  72°C 7 min (final extension) | [5] |
|  | R17K31F: GCTCTTGCAGCTTCTATGTTACA  Rr2608Rnew: CATTGTCCGTCAGGTTGGCG | 434 | The 25μL total reaction volume includes Platinum® Taq DNA polymerase (0.1μL), 10x PCR Buffer (2.5μL), 10mM dNTP mixture (0.5μL), 50 mM MgCl2 (1μL), 10 µM primers (0.75μL each), H_2_O, primary PCR product (1μL) | Secondary PCR: 94°C 1 min  35 cycles of: 94°C 30 s, 58°C 30 s, 68°C 1.30 min  72°C 7 min (final extension)  1.2% agarose gel |  |
| *16S rRNA* cPCR | Ehr-16S_F: GGTACCYACAGAAGAAGTCC  Ehr-16S_R: TAGCACTCATCGTTTACAGC | 345 | The 25μL total reaction volume includes 2x Platinum Quantitative PCR SuperMix UDG (12.5μL), primer mix 200nM each (1.5μL), H_2_O, DNA (3μL) | 50°C 2 min, 95°C 8 min,  40 cycles of: 95°C 30 s, 61°C 45 s, 72°C 45s  72°C 2 min (final extension)  1.5% agarose gel | [6,7] |

**References**

1. Calvani NED, Bell L, Carney A, De La Fuente C, Stragliotto T, Tunstall M, et al. The molecular identity of fleas (Siphonaptera) carrying Rickettsia felis, Bartonella clarridgeiae and Bartonella rochalimae from dogs and cats in Northern Laos. Heliyon. 2020;6(7):e04385. doi: 10.1016/j.heliyon.2020.e04385. PMID: 32695906

2. Folmer O, Black M, Hoeh W, Lutz R, Vrijenhoek R. DNA primers for amplification of mitochondrial cytochrome c oxidase subunit I from diverse metazoan invertebrates. Mol Mar Biol Biotechnol. 1994;3(5):294-9. PMID: 7881515.

3. Potiwat R, Sungvornyothin S., Samung Y., Payakkapol A., & Apiwathnasorn C. Identification Of bat ectoparasite Leptocimex Inordinatus from bat-dwelling cave, Kanchanaburi province, Thailand. The Southeast Asian journal of tropical medicine and public health. 2016;47(1):16–22

4. Jiang J, Chan TC, Temenak JJ, Dasch GA, Ching WM, Richards AL. Development of a quantitative real-time polymerase chain reaction assay specific for Orientia tsutsugamushi. Am J Trop Med Hyg. 2004;70(4):351-6. PMID: 15100446

5. Taylor AJ, Vongphayloth K., Vongsouvath M., Grandadam M., Brey PT., Newton PN., et al. Large-Scale Survey for Tickborne Bacteria, Khammouan Province, Laos. Emerging infectious diseases. 2016; 22 (9):1635–9. doi: <https://doi.org/10.3201/eid2209.151969>.

6. Dao TTH, Takacs N, Tran TN, Truong AN, Skinner K, Kontschan J, et al. Detection of tick-borne pathogens in the pangolin tick, Amblyomma javanense, from Vietnam and Laos, including a novel species of Trypanosoma. Acta Trop. 2024;260:107384. Epub 2024/09/13. doi: 10.1016/j.actatropica.2024.107384. PubMed PMID: 39265756.

7. Parola P, Roux V, Camicas JL, Baradji I, Brouqui P, Raoult D. Detection of ehrlichiae in African ticks by polymerase chain reaction. Trans R Soc Trop Med Hyg. 2000;94(6):707-8. doi: 10.1016/s0035-9203(00)90243-8. PMID: 11198664
