# Supplementary figures and images for: Identification and pathogen screening of ectoparasites from companion animals in urban Vientiane, Lao PDR
Source: PLoS Negl Trop Dis. 2025 Oct 15;19(10):e0013625. doi: 10.1371/journal.pntd.0013625 (PMC12543278; doi:10.1371/journal.pntd.0013625)

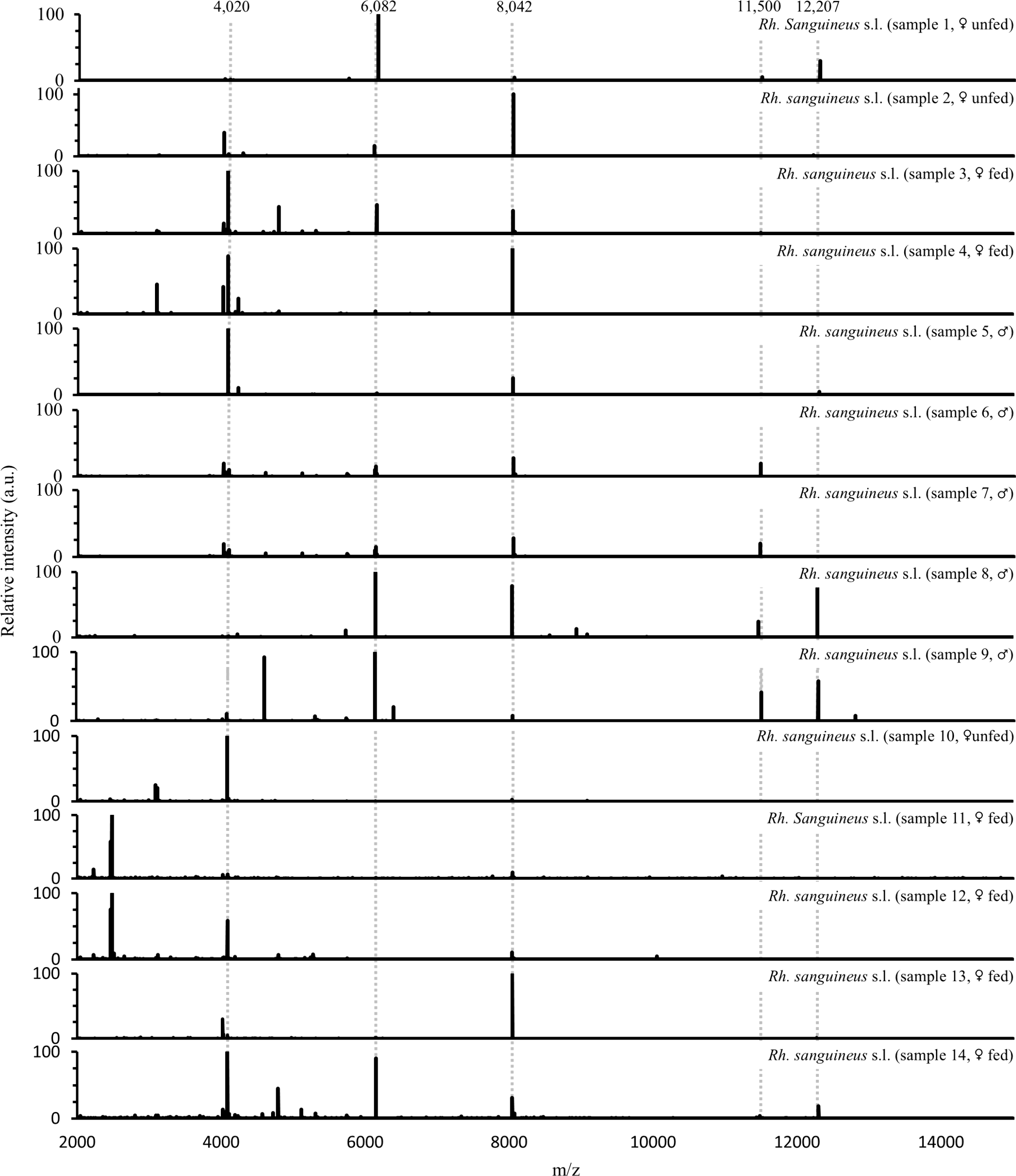

Supplement: S1 Fig — Grey dotted lines represent key identifying peaks from previously published Rh. sanguineus spectra. (TIF) [file pntd.0013625.s005.tif]
